# Stroke Risk Following Nonarteritic Anterior Ischemic Optic Neuropathy

**DOI:** 10.1001/jamanetworkopen.2024.44534

**Published:** 2024-11-12

**Authors:** Yung-Yu Chu, Chung-Han Ho, Yi-Chen Chen, Shu-Chun Kuo

**Affiliations:** 1 Department of Ophthalmology, Chi Mei Medical Center, Tainan, Taiwan; 2 Department of Medical Research, Chi Mei Medical Center, Tainan, Taiwan; 3 Department of Information Management, Southern Taiwan University of Science and Technology, Tainan, Taiwan; 4 Department of Optometry, Chung Hwa University of Medical Technology, Tainan, Taiwan

## Abstract

**Importance:**

The association between nonarteritic anterior ischemic optic neuropathy (NAION) and an increased risk of stroke has been a subject of debate. However, multinational studies on this topic are scarce.

**Objective:**

To evaluate the short-term and long-term stroke risk after NAION compared with a matched control group.

**Design, Setting, and Participants:**

This global, retrospective, population-based cohort study used aggregated electronic health records from January 1, 2004, through March 19, 2024, sourced from the Global Collaborative Network of TriNetX, which includes data from over 152 million patients across 17 countries. Patients in the study were followed up for a maximum duration of 10 years. Patients with NAION and age-related cataract were included in the analysis. Those with stroke before the diagnosis of NAION and age-related cataract were excluded. Propensity score matching was applied to balance age, sex, race, ethnicity, comorbidities, and medication use.

**Exposure:**

*International Statistical Classification of Diseases and Related Health Problems, Tenth Revision (ICD-10)* diagnosis code for NAION or age-related cataract.

**Main Outcomes and Measures:**

The primary outcome was the relative risk (RR) of stroke (*ICD-10* code I60-63) in the NAION cohort vs the matched controls. Multivariable logistic regression analyses were applied to identify potential clinical factors associated with stroke within the NAION cohort.

**Results:**

A total of 89 811 patients were identified in both the NAION (mean [SD] age, 57.2 [18.5] years; 38 678 men [43.1%]) and control (mean [SD] age, 57.0 [17.9] years; 40 014 men [44.6%]) cohorts after matching. The NAION cohort demonstrated a significantly higher all-stroke risk at all time points: 1 month (RR, 5.04; 95% CI, 4.41-5.78), 3 months (RR, 3.79; 95% CI, 3.40-4.21), 1 year (RR, 2.50; 95% CI, 2.32-2.70), 5 years (RR, 1.54; 95% CI, 1.45-1.63), and 10 years (RR, 1.33; 95% CI, 1.23-1.43). Sensitivity analysis in patients without comorbidities similarly revealed a significantly increased all-stroke risk across all intervals: 1 month (RR, 7.55; 95% CI, 4.74-12.03), 3 months (RR, 6.70; 95% CI, 4.48-10.04), 1 year (RR, 3.96; 95% CI, 2.94-5.34), 5 years (RR, 2.85; 95% CI, 2.18-3.72), and 10 years (RR, 1.68; 95% CI, 1.25-2.26). Among all the clinical factors of interest, only hypertension was consistently associated with all subtypes of stroke following NAION.

**Conclusions and Relevance:**

This cohort study of patients with NAION found a significantly elevated risk of stroke compared with matched controls, independently of comorbidities. These findings underscore the importance of regular stroke workups following the onset of NAION.

## Key Points

**Question:**

Is the risk of stroke increased in patients experiencing nonarteritic anterior ischemic optic neuropathy (NAION) compared with a matched control population?

**Findings:**

This multinational cohort study of 89 811 patients with NAION found a significantly elevated risk of stroke following NAION compared with 89 811 propensity score–matched patients without NAION, over both short-term and long-term periods, irrespective of comorbidities.

**Meaning:**

These findings suggest the possible necessity for regular stroke workup following the onset of NAION.

## Teaser

This multinational cohort study examines short-term and long-term stroke risk among patients with nonarteritic anterior ischemic optic neuropathy compared with a matched control group.

## Introduction

Nonarteritic anterior ischemic optic neuropathy (NAION) is characterized by abrupt, painless vision loss and is one of the leading causes of blindness in adults.^1^ It is the most frequently occurring acute optic neuritis in those older than 50 years and is second only to glaucoma in overall optic neuropathy prevalence.^2^ The multifaceted pathophysiology of NAION is not entirely elucidated, but the hypoperfusion of the small arteries and arterioles supplying the optic nerve head is believed to be a primary factor.^1^ Incidence rates of NAION are reported to range from 2.3 to 82 cases per 100 000 person-years,^1,2,3,4^ predominantly affecting older adults with a spectrum of systemic risk factors.^1,5^ Interestingly, a number of risk factors are shared between NAION and stroke, with notable ones being diabetes, obstructive sleep apnea, smoking, dyslipidemia, hypertension, and cardiovascular diseases.^5,6,7,8^ This association has prompted investigations into the potential elevation of stroke risk in patients with NAION. The inquiry into the association between NAION and stroke risk, however, has yielded inconclusive and varied findings.^3,9,10,11,12,13,14^ Some studies^3,9,10^ indicate a considerably higher risk of stroke in patients with NAION with comorbid conditions, yet the extent of risk across the whole NAION population is still a matter of debate.

Notwithstanding the volume of research conducted on the subject, the lack of multinational studies represents a noteworthy limitation. Predominantly, the extant literature is limited to single-center^9,10,12,14^ or single-country^3,11,13^ investigations, often with racially homogeneous participant cohorts.^3,11,13,14^ To bridge the extant research gap, our study seeks to use TriNetX, an international, aggregated electronic medical record database, to evaluate the stroke risk after NAION in comparison with a matched control population.

## Methods

### Study Population

This study used data from the TriNetX Research Network, which contains real-time information on more than 250 million patients from 127 health care organizations. The data used in this study were collected on July 30, 2024. We focused on the Global Collaborative Network, comprising 127 health care organizations across 17 countries with 152 387 781 patients. This study was approved by the institutional review board of the Chi Mei Medical Center and conducted under the principles of the Declaration of Helsinki.^15^ The necessity for obtaining informed consent was exempted given that the study was based exclusively on aggregated data and statistical summaries derived from deidentified information, in accordance with 45 CFR §46. No protected health information was accessed, and no study-specific interventions were conducted in this retrospective analysis. This research adhered to the Strengthening the Reporting of Observational Studies in Epidemiology (STROBE) reporting guidelines, ensuring compliance with reporting standards.^16^

### Study Design

This retrospective cohort study was conducted over a 20-year interval, commencing on January 1, 2004, and concluding on March 19, 2024. Inclusion criteria encompassed all incident cases of NAION identified within the time frame of January 1, 2004, to March 19, 2023, thereby ensuring a minimum of 1-year follow-up after diagnosis for each patient. Mortality within the first year after diagnosis did not preclude data inclusion in the study’s analysis.

For the identification of incident NAION cases, we used the H47.0 diagnostic code in accordance with the *International Statistical Classification of Diseases and Related Health Problems, Tenth Revision (ICD-10)*, which was also used in other studies.^11,17^ The index date was determined as the date of the earliest claim using the NAION diagnostic code.

Exclusion criteria were defined to omit patients with conditions that could potentially be confounded with NAION throughout the study period. This included patients with diagnoses of giant cell arteritis (*ICD-10* codes M31.5 and 31.6), polymyalgia rheumatica (*ICD-10* code M35.5), multiple sclerosis (*ICD-10* code G35), optic neuritis (*ICD-10* code H46), and other demyelinating diseases of the central nervous system (*ICD-10* code G36) including neuromyelitis optica. Patients with a stroke (*ICD-10* codes I60-63) before the index date were likewise excluded. Subsequent to applying these criteria, a cohort of 104 996 patients with NAION diagnosed between 2004 and 2023 was established for analysis.

Baseline characteristics such as age at event, race, ethnicity, and sex were documented. Race and ethnicity data were included in our study as potential covariates and were reported by the health care institutions collaborating with the TriNetX platform. Furthermore, an assessment of comorbidities and comedication usage was conducted to identify potential confounders in the association between NAION and subsequent stroke risk. The presence of comorbidities and the usage of comedications were determined on the basis of historical diagnoses and prescription records up to 2 years before the index date. Comorbidities of interest included diabetes, obstructive sleep apnea, dyslipidemia, hypertension, heart failure, atrial fibrillation and/or flutter, ischemic heart diseases, chronic kidney disease, morbid obesity, tobacco use, and alcohol-related disorder. In addition, specific cerebrovascular diseases, such as cerebral aneurysm, vascular malformations (including arteriovenous malformation, dural arteriovenous fistula, and others), and steno-occlusive vasculopathy, were also included. Furthermore, conditions associated with elevated homocysteine levels, including vitamin B_12_ deficiency and disorders of sulfur-bearing amino acid metabolism, were identified (see eTable 1 in Supplement 1 for relevant *ICD-10* codes). Information on comedication encompassed the use of antiplatelet agents, anticoagulant agents, antilipemic agents, oral hypoglycemic agents, insulin, α-blockers, β-blockers, calcium channel blockers, diuretics, angiotensin-converting enzyme inhibitors, and angiotensin II receptor blockers (see eTable 1 in Supplement 1 for detailed drug codes).

A control group was subsequently established, consisting of patients with initial diagnoses of age-related cataracts between January 1, 2004, and March 19, 2023. This ensured that the control cases also had at least 1 year of follow-up. If patients died within 1 year, they were still included in the analysis. To identify the controls with age-related cataracts, we used the *ICD-10* code H25. Patients who had experienced a stroke before the diagnosis of age-related cataracts were excluded. Furthermore, any patient in the cataract control group was excluded if they experienced an NAION event during the study period. On the basis of these criteria, 2 001 698 patients with age-related cataract diagnosed between 2004 and 2023 were included.

### Main Outcomes

The primary outcome of this study was to assess the rate of all stroke (*ICD-10* codes I60-63) at various time intervals: 1 month, 3 months, 1 year, 5 years, and 10 years. Furthermore, the rates of ischemic stroke (*ICD-10* code I63) and hemorrhagic stroke (*ICD-10* codes I60-62) were separately quantified at these identical time points. Analyses at the 5-year and 10-year marks were restricted to patients with a minimum corresponding follow-up period. Cases of mortality within these intervals were not excluded from the analysis. The relative risk (RR) and risk difference (RD) were calculated to compare the risk of outcomes between the NAION cohort and the cataract control group.

### Statistical Analysis

Statistical analyses were performed using SPSS statistical software version 19 (IBM) and the built-in statistical function of the TriNetX network. Propensity score matching (PSM) was used to equilibrate baseline characteristics such as age, sex, race, ethnicity, the aforementioned comorbidities, and comedications between the NAION and cataract control cohorts. We used a logistic regression model to optimize the variates of the 2 cohorts, and the closest propensity scores were estimated. Matching (1:1) by nearest-neighbor greedy matching algorithm with a caliper of 0.1 SD was used to derive matched pairs. The propensity score, denoting the likelihood of exposure to either NAION or cataract control patients, was determined using the baseline characteristics of age, sex, race, ethnicity, the aforementioned comorbidities, and comedications adjusted into the regression model.^18^ The use of nearest-neighbor greedy matching with a caliper width of 0.1 pooled SD algorithm imposes a maximum difference in propensity scores between NAION and cataract control patients within a matched pair, leading to less-biased estimates than other matching algorithms.^19^ Standardized mean difference (SMD) was performed to analyze the homogeneity of category variables, including age, sex, race, ethnicity, comorbidities, and comedications between the NAION and cataract control cohorts. SMD greater than 0.1 indicates a significant difference between the 2 groups.^20^ Considering the presence of 2 types of stroke, the Bonferroni correction was used to adjust for multiple comparisons. *P *< .025 was considered statistically significant.

#### Risk Factor Analysis

Risk factor analysis was conducted to evaluate the association between clinical factors of interest and stroke within the NAION cohort. These clinical factors included age, sex, and comorbidities. Logistic regressions using multivariable analyses were applied.

#### Sensitivity Analysis

To discern the isolated association of NAION with stroke risk, a sensitivity analysis was performed of patients without comorbidities. This involved excluding those with any diagnostic claim for the aforementioned comorbidities during the study period from both cohorts. Following this refinement, PSM with 1:1 matching by age, sex, race, ethnicity, and the aforementioned comedications was conducted for the remaining patients within both cohorts. Rates of all stroke, ischemic stroke, and hemorrhagic stroke were then evaluated at the same time intervals specified in the primary analysis. Consistent with the primary analysis, only patients with at least 5 or 10 years of follow-up were included in the analyses performed at the corresponding intervals, whereas cases of mortality within these periods were retained in the analysis. RR and RD were also calculated for each interval.

## Results

### Baseline Demographic and Clinical Characteristics

A total of 89 811 patients were included in both the NAION (mean [SD] age, 57.2 [18.5] years; 49 700 women [55.3%]; 38 678 men [43.1%], 1433 unknown [1.6%]) and cataract control (mean [SD] age, 57.0 [17.9] years; 47 954 women [55.4%]; 40 014 men [44.6%], 1843 unknown [2.1%]) cohorts after PSM. Race distribution in the NAION cohort was 0.3% American Indian or Alaska Native (268 individuals), 4.4% Asian (3965 individuals), 11.5% Black or African American (10 326 individuals), 0.6% Native Hawaiian or Other Pacific Islander (571 individuals), 55.4% White (49 739 individuals), 3.3% other race (which includes multiracial and any other race not specified; 2937 individuals), and 24.5% unknown (22 005 individuals). In the cataract control cohort, race distribution was similar, with 0.3% American Indian or Alaska Native (277 individuals), 4.2% Asian (3809 individuals), 11.5% Black or African American (10 306 individuals), 0.8% Native Hawaiian or Other Pacific Islander (744 individuals), 55.2% White (49 598 individuals), 3.5% other race (3144 individuals), and 24.4% unknown (21 933 individuals). Ethnicity in the NAION cohort was 7.0% Hispanic or Latino (6278 individuals), 70.1% not Hispanic or Latino (62 982 individuals), and 22.9% unknown (20 551 individuals); in the control cohort, ethnicity was 6.9% Hispanic or Latino (6208 individuals), 69.3% not Hispanic or Latino (62 242 individuals), and 23.8% unknown (21 361 individuals).

A flowchart detailing the inclusion and allocation processes is shown in the Figure. Table 1 presents the baseline characteristics and underlying conditions of the NAION cohort and cataract control cohort before and after the PSM. After PSM, the SMD for all variables was less than 0.1, suggesting the achievement of balanced cohorts.

**Figure.**
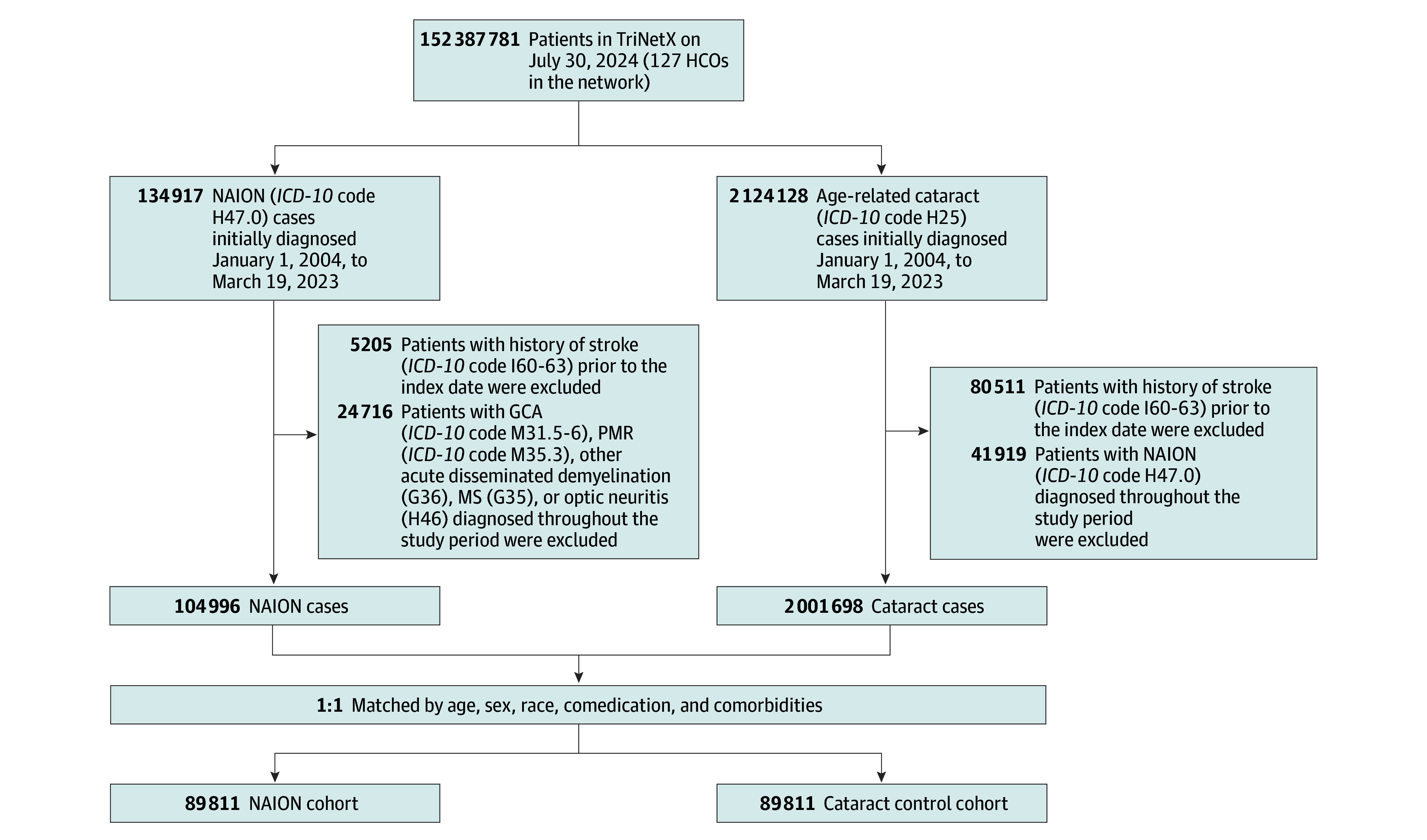
Flowchart of the Selection of Study Participants GCA indicates giant cell arteritis; HCO, health care organization; *ICD-10, International Statistical Classification of Diseases and Related Health Problems, Tenth Revision*; MS, multiple sclerosis; NAION, nonarteritic anterior ischemic optic neuropathy; and PMR, polymyalgia rheumatica.

**Table 1.** Baseline Characteristics of NAION Cohort and Cataract Control Cohort Before and After PSM

Characteristic	Before PSM			After PSM		
	Participants, No. (%)		SMD	Participants, No. (%)		SMD
	NAION cohort (n = 104 996)	Cataract control cohort (n = 2 001 698)		NAION cohort (n = 89 811)	Cataract control cohort (n = 89 811)	
Age at index, mean (SD), y	50.1 (24.5)	66.5 (10.7)	0.87^a^	57.2 (18.5)	57.0 (17.9)	0.01
Sex						
Female	57 209 (54.5)	1 096 879 (54.8)	0.01	49 700 (55.3)	47 954 (55.4)	0.04
Male	46 332 (44.1)	817 497 (40.8)	0.07	38 678 (43.1)	40 014 (44.6)	0.03
Unknown	1455 (1.4)	87 322 (4.4)	0.18^a^	1433 (1.6)	1843 (2.1)	0.03
Race						
American Indian or Alaska Native	324 (0.3)	6396 (0.3)	0.002	268 (0.3)	277 (0.3)	0.002
Asian	4434 (4.2)	68 256 (3.4)	0.04	3965 (4.4)	3809 (4.2)	0.01
Black or African American	11 841 (11.3)	235 998 (11.8)	0.02	10 326 (11.5)	10 306 (11.5)	0.001
Native Hawaiian or Other Pacific Islander	597 (0.6)	32 779 (1.6)	0.10^a^	571 (0.6)	744 (0.8)	0.02
White	58 654 (55.9)	1 118 857 (55.5)	0.001	49 739 (55.4)	49 598 (55.2)	0.003
Other^b^	3894 (3.7)	68 368 (3.4)	0.02	2937 (3.3)	3144 (3.5)	0.01
Unknown	25 252 (24.1)	471 044 (23.5)	0.01	22 005 (24.5)	21 933 (24.4)	0.002
Ethnicity						
Hispanic or Latino	8404 (8.0)	127 307 (6.4)	0.06	6278 (7.0)	6208 (6.9)	0.003
Not Hispanic or Latino	74 004 (70.5)	1 212 130 (60.6)	0.21^a^	62 982 (70.1)	62 242 (69.3)	0.02
Unknown	22 588 (21.5)	646 032 (33.1)	0.26^a^	20 551 (22.9)	21 361 (23.8)	0.02
Comorbidities						
Diabetes	16 797 (16.0)	516 195 (25.7)	0.24^a^	16 745 (18.6)	17 248 (19.2)	0.01
Dyslipidemia	23 886 (22.7)	693 725 (34.7)	0.27^a^	23 652 (26.3)	23 586 (26.3)	0.002
Hypertension	26 967 (25.7)	801 266 (40.0)	0.31^a^	26 694 (29.7)	27 088 (30.2)	0.01
Heart failure	3327 (3.2)	85 607 (4.3)	0.06	3236 (3.6)	3309 (3.7)	0.004
Atrial fibrillation and/or flutter	3709 (3.5)	96 300 (4.8)	0.06	3695 (4.1)	3635 (4.0)	0.003
Ischemic heart diseases	7542 (7.2)	210 147 (10.5)	0.12^a^	7509 (8.4)	7437 (8.3)	0.003
Chronic kidney disease	5139 (4.9)	128 754 (6.4)	0.07	5018 (5.6)	5159 (5.7)	0.01
Tobacco use	4239 (4.0)	117 683 (5.9)	0.09	4221 (4.7)	4256 (4.7)	0.002
Obstructive sleep apnea	5079 (4.8)	110 824 (5.5)	0.03	4660 (5.2)	4286 (4.8)	0.02
Arteriovenous malformation of cerebral vessels	127 (0.1)	548 (0.03)	0.03	81 (0.1)	89 (0.1)	0.003
Other malformations of cerebral vessels	170 (0.2)	752 (0.04)	0.04	106 (0.1)	111 (0.1)	0.002
Nonruptured cerebral aneurysm	561 (0.5)	4218 (0.2)	0.05	519 (0.6)	495 (0.6)	0.004
Occlusion and stenosis of precerebral arteries	1862 (1.8)	31 364 (1.6)	0.02	1829 (2.0)	1561 (1.7)	0.02
Occlusion and stenosis of cerebral arteries	158 (0.2)	1227 (0.1)	0.03	128 (0.1)	132 (0.1)	0.001
Dural arteriovenous fistula	139 (0.1)	1669 (0.1)	0.02	130 (0.1)	127 (0.1)	0.001
Morbid obesity	3133 (3.0)	73 516 (3.7)	0.04	3071 (3.4)	2831 (3.2)	0.02
Disorders of sulfur-bearing amino acid metabolism	94 (0.1)	1224 (0.1)	0.01	84 (0.1)	99 (0.1)	0.01
Vitamin B_12_ deficiency	379 (0.4)	9681 (0.5)	0.02	377 (0.4)	405 (0.5)	0.01
Alcohol-related disorder	1165 (1.1)	31 156 (1.6)	0.04	1160 (1.3)	1175 (1.3)	0.001
Comedication						
Antiplatelet agents	10 860 (10.3)	263 258 (13.2)	0.09	10 721 (11.9)	10 298 (11.5)	0.02
Anticoagulant agents	9462 (9.0)	188 035 (9.4)	0.01	8933 (9.9)	8357 (9.3)	0.02
Antilipemic agents	17 230 (16.4)	474 230 (23.7)	0.18^a^	17 167 (19.1)	16 994 (18.9)	0.01
Oral hypoglycemic agents	8309 (7.9)	224 529 (11.2)	0.11^a^	8263 (9.2)	8135 (9.1)	0.01
Insulin	6184 (5.9)	136 926 (6.8)	0.04	6121 (6.8)	5763 (6.4)	0.02
α-Blockers	3469 (3.3)	87 409 (4.4)	0.06	3444 (3.8)	3227 (3.6)	0.01
β-Blockers	15 172 (14.5)	346 190 (17.3)	0.08	14 872 (16.6)	15 089 (16.8)	0.01
Calcium channel blockers	9438 (9.0)	236 628 (11.8)	0.09	9339 (10.4)	9189 (10.2)	0.01
Diuretics	13 839 (13.2)	344 025 (17.2)	0.11^a^	13 244 (14.7)	13 117 (14.6)	0.004
Angiotensin-converting enzyme inhibitors	8825 (8.4)	265 486 (13.3)	0.16^a^	8735 (9.7)	9199 (10.2)	0.02
Angiotensin II inhibitors	6449 (6.1)	172 954 (8.6)	0.10	6431 (7.2)	6275 (7.0)	0.01

Abbreviations: NAION, nonarteritic anterior ischemic optic neuropathy; PSM, propensity score matching; SMD, standardized mean difference.

^a^ Statistically significant at SMD greater than 0.1.

^b^ Includes multiracial and any other race not specified.

### Main Outcomes

Table 2 shows the rate of all stroke, ischemic stroke, and hemorrhagic stroke in the NAION group compared with the cataract control group. There were 56 237 patients in the NAION cohort and 22 761 patients in the control cohort with at least 5 or 10 years of follow-up. The NAION cohort exhibited a significantly higher risk of all stroke (1 month, RR, 5.04 [95% CI, 4.41-5.78]; 3 months, RR, 3.79 [95% CI, 3.40-4.21]; 1 year, RR, 2.50 [95% CI, 2.32-2.70]; 5 years, RR, 1.54 [95% CI, 1.45-1.63]; and 10 years, RR, 1.33 [95% CI, 1.23-1.43]), ischemic stroke, and hemorrhagic stroke across all follow-up intervals, although the relative RD for hemorrhagic stroke was modest at these time points.

**Table 2.** Rate of All Stroke, Hemorrhagic Stroke, and Ischemic Stroke After NAION Compared With the Cataract Control Cohort in a Propensity Score–Matched Analysis

Follow-up interval and stroke type	RR (95% CI)	RD, % (95% CI)	Patients who experienced stroke, No. (%)		*P* value^a^
			NAION cohort	Control cohort	
1-mo Follow-up					
No. of patients	NA	NA	89 811	89 811	NA
All stroke	5.04 (4.41-5.78)	1.14 (1.05-1.22)	1276 (1.42)	253 (0.28)	<.001
Hemorrhagic stroke	7.49 (5.56-10.09)	0.35 (0.31-0.40)	367 (0.41)	49 (0.06)	<.001
Ischemic stroke	4.68 (4.03-5.42)	0.87 (0.80-0.95)	996 (1.11)	213 (0.24)	<.001
3-mo Follow-up					
No. of patients	NA	NA	89 811	89 811	NA
All stroke	3.79 (3.40-4.21)	1.31 (1.21-1.41)	1598 (1.78)	422 (0.47)	<.001
Hemorrhagic stroke	5.25 (4.18-6.58)	0.42 (0.37-0.47)	467 (0.52)	89 (0.10)	<.001
Ischemic stroke	3.53 (3.14-3.98)	0.99 (0.90-1.07)	1237 (1.38)	350 (0.39)	<.001
1-y Follow-up					
No. of patients	NA	NA	89 811	89 811	NA
All stroke	2.50 (2.32-2.70)	1.54 (1.42-1.67)	2312 (2.57)	925 (1.03)	<.001
Hemorrhagic stroke	3.20 (2.74-3.73)	0.51 (0.45-0.58)	668 (0.74)	209 (0.23)	<.001
Ischemic stroke	2.35 (2.16-2.56)	1.15 (1.04-1.26)	1801 (2.01)	765 (0.85)	<.001
5-y Follow-up					
No. of patients	NA	NA	56 237	56 237	NA
All stroke	1.54 (1.45-1.63)	1.74 (1.50-1.97)	2797 (4.97)	1821 (3.24)	<.001
Hemorrhagic stroke	1.65 (1.48-1.85)	0.56 (0.43-0.68)	791 (1.41)	478 (0.85)	<.001
Ischemic stroke	1.50 (1.41-1.60)	1.33 (1.12-1.54)	2244 (3.99)	1495 (2.66)	<.001
10-y Follow-up					
No. of patients	NA	NA	22 761	22 761	NA
All stroke	1.33 (1.23-1.43)	1.73 (1.29-2.17)	1598 (7.02)	1204 (5.29)	<.001
Hemorrhagic stroke	1.38 (1.20-1.59)	0.54 (0.30-0.78)	450 (1.98)	327 (1.44)	<.001
Ischemic stroke	1.30 (1.20-1.41)	1.33 (0.93-1.73)	1294 (5.69)	992 (4.36)	<.001

Abbreviations: NA, not applicable; NAION, nonarteritic anterior ischemic optic neuropathy; RD, risk difference; RR, relative risk.

^a^ *P* values were calculated for RD. All *P* values were statistically significant at *P* < .025 after the Bonferroni correction.

### Risk Factor Analysis

eTable 2 in Supplement 1 details the risk factor analysis result. In summary, among all clinical factors of interest, only hypertension was consistently associated with all, hemorrhagic, and ischemic stroke within 1 and 10 years after NAION.

### Sensitivity Analysis

After excluding patients with comorbidities, the NAION cohort consisted of 42 546 patients, and the cataract control cohort included 416 628 patients. A total of 29 927 patients were identified in the NAION cohort and cataract control cohort after PSM. The baseline characteristics of the refined NAION cohort and cataract control cohort before and after the PSM are shown in eTable 3 in Supplement 1. After PSM, the SMDs for all variables were less than 0.1, indicating that balanced cohorts were achieved.

Table 3 illustrates the rates of all strokes, ischemic strokes, and hemorrhagic strokes among patients without comorbidities within the NAION group compared with those in the cataract control group. There were 19 232 patients in the NAION cohort and 8326 patients in the control cohort with at least 5 or 10 years of follow-up. Generally, rates of all strokes, ischemic strokes, and hemorrhagic strokes were lower in both cohorts compared with the primary analysis. Nonetheless, consistent with the primary analysis, the risks of all strokes (1 month, RR, 7.55 [95% CI, 4.74-12.03]; 3 months, RR, 6.70 [95% CI, 4.48-10.04]; 1 year, RR, 3.96 [95% CI, 2.94-5.34]; 5 years, RR, 2.85 [95% CI, 2.18-3.72]; and 10 years, RR, 1.68 [95% CI, 1.25-2.26]), ischemic strokes, and hemorrhagic strokes following NAION were significantly higher at all the follow-up intervals. The magnitude of the RD, however, was relatively small at all these time points.

**Table 3.** Comparative Rates of All Stroke, Hemorrhagic Stroke, and Ischemic Stroke Among Patients Without Comorbidities Following NAION vs a Propensity Score–Matched Cataract Control Cohort

Follow-up interval and stroke type	RR (95% CI)	RD, % (95% CI)	Patients who experienced stroke, No. (%)		*P* value^a^
			NAION cohort	Control cohort	
1-mo Follow-up					
No. of patients	NA	NA	29 927	29 927	NA
All stroke	7.55 (4.74-12.03)	0.44 (0.35-0.52)	151 (0.51)	20 (0.07)	<.001
Hemorrhagic stroke	6.6 (3.34-12.83)	0.19 (0.13-0.24)	66 (0.22)	10 (0.03)	<.001
Ischemic stroke	8.27 (4.43-15.46)	0.27 (0.20-0.33)	91 (0.30)	11 (0.04)	<.001
3-mo Follow-up					
No. of patients	NA	NA	29 927	29 927	NA
All stroke	6.70 (4.48-10.04)	0.52 (0.42-0.61)	181 (0.61)	27 (0.09)	<.001
Hemorrhagic stroke	7.5 (3.88-14.51)	0.22 (0.16-0.28)	75 (0.25)	10 (0.03)	<.001
Ischemic stroke	6.22 (3.78-10.23)	0.31 (0.24-0.39)	112 (0.37)	18 (0.06)	<.001
1-y Follow-up					
No. of patients	NA	NA	29 927	29 927	NA
All stroke	3.96 (2.94-5.34)	0.54 (0.43-0.64)	214 (0.72)	54 (0.18)	<.001
Hemorrhagic stroke	4.53 (2.76-7.44)	0.22 (0.16-0.29)	86 (0.29)	19 (0.06)	<.001
Ischemic stroke	3.72 (2.58-5.38)	0.33 (0.24-0.41)	134 (0.45)	36 (0.12)	<.001
5-y Follow-up					
No. of patients	NA	NA	19 232	19 232	NA
All stroke	2.85 (2.18-3.72)	0.69 (0.52-0.86)	205 (1.07)	72 (0.37)	<.001
Hemorrhagic stroke	3.39 (2.13-5.40)	0.29 (0.18-0.39)	78 (0.41)	23 (0.12)	<.001
Ischemic stroke	2.56 (1.86-3.52)	0.42 (0.28-0.56)	133 (0.69)	52 (0.27)	<.001
10-y Follow-up					
No. of patients	NA	NA	8326	8326	NA
All stroke	1.68 (1.25-2.26)	0.56 (0.25-0.88)	116 (1.39)	69 (0.83)	<.001
Hemorrhagic stroke	1.94 (1.08-3.48)	0.19 (0.03-0.36)	33 (0.40)	17 (0.20)	.02
Ischemic stroke	1.52 (1.09-2.12)	0.35 (0.07-0.63)	85 (1.02)	56 (0.67)	.01

Abbreviations: NA, not applicable; NAION, nonarteritic anterior ischemic optic neuropathy; RD, risk difference; RR, relative risk.

^a^ *P* values were calculated for RD. All *P* values were statistically significant at *P* < .025 after the Bonferroni correction.

## Discussion

In this multinational retrospective cohort study, we found a significantly elevated stroke risk among patients with NAION compared with matched controls, over both short-term and long-term periods, irrespective of comorbidities. Several risk factors are shared between NAION and stroke,^1,6^ prompting studies into their association, yet results remain conflicting.^3,9,10,11,12,13,14^ Although some studies^3,9,10^ have reported a significantly elevated stroke risk in patients with NAION with comorbid conditions such as diabetes and hypertension, findings regarding the entire population of patients with NAION are widely disputed. Most previous studies^9,11,12,13^ found no increase in stroke risk among patients with NAION, but Lee et al^3^ identified a significantly elevated ischemic stroke risk following NAION. Li and associates^14^ also found a higher ratio of ischemic stroke in patients with NAION compared with age-matched, sex-matched, and weight-matched controls. However, the controls in both studies were not matched for common comorbidities associated with stroke. Therefore, Hayreh^21^ argued that the increased risk was not directly caused by NAION but by the associated comorbidities. Although our study’s findings also concur with those of Lee et al^3^ and Li et al,^14^ we further consider the associations of comorbidities with stroke risk. After adjusting for comorbidities and comedications using PSM, our study still revealed a significantly elevated stroke risk following NAION. Furthermore, a sensitivity analysis of patients without comorbidities yielded a similar result. Thus, the findings of our study suggest that NAION is independently associated with increased stroke risk, regardless of the presence of comorbidities.

Hayreh^21^ also posited that NAION is a distinct clinical entity from cerebral ischemic stroke owing to differing underlying pathophysiology, with NAION being a hypotensive disorder and stroke a thromboembolic disorder in most cases. Consequently, he concluded that there is no direct link between NAION and stroke.^21^ Nonetheless, we hypothesized that strokes associated with NAION might have a different cause. Cerebrovascular diseases have 3 primary causes: large artery atherosclerosis, cardioembolism, and small vessel disease (SVD).^22^ Although the first 2 are the most common, SVD still contributes to 25% of ischemic strokes.^23,24^ Increasing evidence supports an association between cerebral SVD and NAION.^13,25,26,27^ In a study reviewing magnetic resonance images of 2812 patients, Kim and associates^25^ found a significantly higher ratio of cerebral SVD in patients with NAION than in controls after adjusting for age, sex, and comorbidities. Foster and associates^13^ also reported a significantly higher proportion of cerebral SVD in patients with NAION compared with controls matched for age, sex, and vascular risk factors. Therefore, these findings may indicate that NAION is associated with SVD, regardless of comorbidities. Compromised cerebral blood flow and cerebrovascular reactivity from arteriolar endothelial dysfunction are central to cerebral SVD pathophysiology.^24^ Given that hypoperfusion in short posterior ciliary arteries underlies NAION,^1,28,29,30^ and both conditions involve arterioles, a pathophysiological link is conceivable. Since cerebral SVD can cause brain damage and stroke,^24^ the elevated stroke risk in our study may also be attributed to cerebral SVD, beyond large artery atherosclerosis or cardioembolism. Still, further investigation is needed to clarify the connection between NAION and cerebral SVD.

The association between hemorrhagic stroke and NAION has been underexplored because of its rarity compared with ischemic stroke. Previous studies, such as those by Lee et al^3^ and Park et al,^31^ found no significant difference in hemorrhagic stroke risk between patients with NAION and controls. However, our study identified a significantly higher risk of hemorrhagic stroke in patients with NAION, regardless of comorbidities. This discrepancy may be attributed to the larger sample size and the broader racial diversity of our study population, which included patients from 17 countries, compared with the smaller, more racially homogeneous populations of earlier studies.^3,31^ Moreover, the significantly elevated hemorrhagic stroke risk noted in our research could also be linked to the association between cerebral SVD and NAION,^13,25^ considering that SVD is responsible for approximately 80% of nontraumatic intracerebral hemorrhages.^32^ We hypothesize that NAION can be considered a variant of SVD affecting the optic nerve, because both conditions share pathophysiological mechanisms involving compromised blood flow and vascular autoregulation at the arteriolar level.^1,24,28,29,30^ Consequently, the presentation of NAION may indicate that patients also exhibit some degree of SVD in their brain. This hypothesis is supported by previous studies^13,25^ that have demonstrated a significantly higher ratio of cerebral SVD in patients with NAION. Given that the burden of SVD is associated with an increased risk of subsequent hemorrhagic stroke,^33^ patients with NAION are, therefore, at an elevated risk of experiencing hemorrhagic strokes. The RD of hemorrhagic stroke between the NAION and control groups was relatively modest, likely owing to the rarity of hemorrhagic stroke compared with ischemic stroke, which accounted the majority of all strokes in our study. Still, the RD was statistically significant in both the primary and sensitivity analyses across all follow-up intervals. Therefore, our findings suggest that NAION is independently associated with increased risk of hemorrhagic stroke, regardless of comorbidities.

This study’s strengths include its large sample size, encompassing diverse data from global health care systems, making it the first, to our knowledge, multinational investigation of its kind. The unprecedented scale ensures statistically robust conclusions, with broad applicability owing to its international scope. A closely matched control population minimized the impact of comorbidities, and a sensitivity analysis of patients without comorbidities clarified NAION’s isolated impact. Finally, as far as we know, this is the only study that examines the hemorrhagic risk after NAION with a robust sample size.

### Limitations

This study also has limitations. A primary concern is the potential for misclassification bias, stemming from the reliance on accurate *ICD-10* diagnosis codes for identifying outcomes and conditions, including NAION. The use of *ICD-10* codes alone limits our ability to assess the detailed workup and diagnostic evaluations performed. In addition, the retrospective nature of the study introduces inherent biases and limitations in data collection. The TriNetX database lacks precise information on metabolic profiles, dietary habits, physical activity levels, alcohol consumption, and the durations of comorbid conditions, all of which are factors that could potentially influence stroke development. Moreover, information on blood pressure, blood glucose levels, and body mass index was not fully available.

## Conclusions

In summary, this multinational cohort study found a significantly elevated stroke rate after NAION over both short-term and long-term periods compared with matched controls, independent of comorbidities. These findings suggest that NAION may be independently associated with increased risk for stroke. Consequently, a regular stroke workup may be necessary following the onset of NAION.

## Notes

Supplement 1. **eTable 1.** Codes used in the study for defining conditions and drugs **eTable 2.** Association between age, sex, comorbidities, and the risk of all strokes, hemorrhagic stroke, and ischemic stroke after non-arteritic anterior ischemic optic neuropathy (adjusted) **eTable 3.** Baseline characteristic of NAION cohort and cataract control among patients without comorbidities before and after PSM

Supplement 2.
Data Sharing Statement
