# Supplementary material for: Stroke Risk Following Nonarteritic Anterior Ischemic Optic Neuropathy
Source: JAMA Netw Open. 2024 Nov 12;7(11):e2444534. doi: 10.1001/jamanetworkopen.2024.44534 (PMC11558474; doi:10.1001/jamanetworkopen.2024.44534)
Supplement: Supplement 1. — eTable 1. Codes used in the study for defining conditions and drugs eTable 2. Association between age, sex, comorbidities, and the risk of all strokes, hemorrhagic stroke, and ischemic stroke after non-arteritic anterior ischemic optic neuropathy (adjusted) eTable 3. Baseline characteristic of NAION cohort and cataract control among patients without comorbidities before and after PSM [file jamanetwopen-e2444534-s001.pdf]

Supplemental Online Content

Chu YY, Ho CH, Chen YC, Kuo SC. Stroke risk following nonarteritic anterior ischemic optic neuropathy. *JAMA Netw Open*. 2024;7(11):e2444534. doi:10.1001/jamanetworkopen.2024.44534

eTable 1. Codes used in the study for defining conditions and drugs
eTable 2. Association between age, sex, comorbidities, and the risk of all strokes, hemorrhagic stroke, and ischemic stroke after non-arteritic anterior ischemic optic neuropathy (adjusted)
eTable 3. Baseline characteristic of NAION cohort and cataract control among patients without comorbidities before and after PSM

This supplemental material has been provided by the authors to give readers additional information about their work.

© 2024 Chu YY et al. *JAMA Network Open.*

eTable 1. Codes used in the study for defining conditions and drugs

Condition	ICD-10 code
**Non-Arteritic Anterior Ischemic Optic Neuropathy**	H47.0
**Diabetes mellitus**	E08-E13
**Dyslipidemia**	E78
**Hypertension**	I10
**Heart failure**	I50
**Atrial fibrillation/flutter**	I48
**Ischemic heart diseases**	I20-I25
**Chronic kidney disease**	N18
**Tobacco use**	F17
**Hemorrhagic Stroke**	I60-I62
**Ischemic stroke**	I63
**Polymyalgia rheumatica**	M35.3
**Giant cell arteritis**	M31.5-M31.6
**Multiple sclerosis**	G35
**Other acute disseminated demyelination**	G36
**Optic neuritis**	H46
**Age-related cataract**	H25
**Obstructive sleep apnea**	G47.33
**Arteriovenous malformation of cerebral vessels**	Q28.2
**Other malformations of cerebral vessels**	Q28.3
**Nonruptured cerebral aneurysm**	I67.1
**Occlusion and stenosis of precerebral arteries**	I65
**Occlusion and stenosis of cerebral arteries**	I66
**Dural arteriovenous fistula**	I77.0
**Morbid obesity**	E66.01
**Disorders of sulfur-bearing amino-acid metabolism**	E72.1
**Vitamin B12 deficiency**	D51
**Alcohol related disorder**	F10
**Drug**	**RxNorm code**
**Antiplatelet agents**	BL117
**Anticoagulant agents**	BL110
**Antilipemic agents**	CV350
**Oral Hypoglycemic agents**	HS502
**Insulin**	HS501
**Alpha blockers**	CV150
**Beta blockers**	CV100
**Calcium channel blockers**	CV200
**Diuretics**	CV700
**Angiotensin-converting enzyme inhibitors**	CV800
**Angiotensin II inhibitors**	CV805

eTable 2. Association between age, sex, comorbidities, and the risk of all strokes, hemorrhagic stroke, and ischemic stroke after non-arteritic anterior ischemic optic neuropathy (adjusted)

Characteristic	All Stroke Adjusted OR (95% CI)[b]	*p-value*	Hemorrhagic Stroke Adjusted OR (95% CI)[b]	*p-value*	Ischemic stroke Adjusted OR (95% CI)[b]	*p-value*
**1 year**						
**Age**	1.01 (1.01, 1.01)	**<.001***	1.00 (0.99, 1)	.05	1.02 (1.02, 1.02)	**<.001***
**Male**	1.28 (1.18, 1.39)	**<.001***	1.35 (1.17, 1.56)	**<.001***	1.21 (1.10, 1.34)	**<.001***
**Diabetes mellitus**	0.99 (0.89, 1.1)	.84	0.85 (0.69, 1.04)	.10	1.04 (0.92, 1.17)	.54
**Dyslipidemia**	0.94	.24	0.92	.40	0.93	.25

© 2024 Chu YY et al. *JAMA Network Open.*

	(0.84, 1.04)		(0.75, 1.12)		(0.82, 1.05)	
**Hypertension**	1.66 (1.49, 1.85)	**<.001***	1.85 (1.52, 2.24)	**<.001***	1.57 (1.39, 1.77)	**<.001***
**Heart failure**	1.41 (1.20, 1.66)	**<.001***	1.45 (1.05, 1.98)	**.02***	1.40 (1.17, 1.67)	**<.001***
**Atrial fibrillation and flutter**	1.56 (1.34, 1.81)	**<.001***	1.59 (1.19, 2.13)	**.002***	1.53 (1.30, 1.80)	**<.001***
**Ischemic heart diseases**	1.58 (1.39, 1.80)	**<.001***	1.38 (1.08, 1.77)	**.01***	1.64 (1.42, 1.88)	**<.001***
**Chronic kidney disease**	1.27 (1.11, 1.47)	**<.001***	1.16 (0.88, 1.53)	.31	1.29 (1.11, 1.51)	**.001***
**Tobacco use**	1.62 (1.39, 1.89)	**<.001***	1.48 (1.12, 1.95)	**.01***	1.67 (1.41, 1.99)	**<.001***
**OSA**	0.89 (0.75, 1.05)	.16	0.89 (0.66, 1.22)	.48	0.866 (0.71, 1.05)	.14
**Alcohol related disorders**	1.15 (0.86, 1.53)	.35	1.24 (0.76, 2.04)	.39	1.11 (0.80, 1.55)	.54
**Nonruptured cerebral aneurysm**	1.37 (0.90, 2.07)	.14	1.78 (0.90, 3.53)	.10	1.28 (0.80, 2.06)	.30
**Occlusion and stenosis of cerebral arteries**	1.21 (0.57, 2.56)	.62	1.20 (0.30, 4.76)	.80	1.22 (0.53, 2.78)	.64
**Occlusion and stenosis of precerebral arteries**	2.02 (1.70, 2.40)	**<.001***	1.41 (0.97, 2.04)	.07	2.31 (1.93, 2.77)	**<.001***
**DAVF**	1.02 (0.48, 2.18)	.96	1.08 (0.28, 4.19)	.91	1.02 (0.44, 2.37)	.97
**AVM of cerebral vessels**	1.02 (0.23, 4.49)	.98	1.08 (0.10, 11.50)	.95	1.02 (0.18, 5.67)	.99
**Other malformations of cerebral vessels**	1.00 (0.27, 3.70)	1	1.03 (0.12, 8.54)	.98	1.00 (0.22, 4.57)	1
**Vitamin B12 deficiency**	1.01 (0.62, 1.65)	.96	1 (0.38, 2.66)	1	1.02 (0.60, 1.72)	.95
**Disorders of sulfur-bearing amino-acid metabolism**	1.00 (0.29, 3.39)	1	0.99 (0.11, 8.78)	.99	1.00 (0.25, 4.00)	1
**Morbid obesity**	0.87 (0.69, 1.09)	.22	0.96 (0.65, 1.42)	.82	0.85 (0.66, 1.11)	.24
**10 year[a]**						
**Age**	1.02 (1.02, 1.03)	**<.001***	1.01 (1.01, 1.02)	**<.001***	1.03 (1.03, 1.03)	**<.001***
**Male**	1.06 (0.95, 1.17)	.32	1.02 (0.85, 1.23)	.82	0.99 (0.88, 1.11)	.80
**Diabetes mellitus**	1.32 (1.15, 1.52)	**<.001***	1.15 (0.90, 1.48)	.27	1.41 (1.21, 1.64)	**<.001***
**Dyslipidemia**	1.21 (1.05, 1.38)	**<.001***	1.12 (0.87, 1.43)	.38	1.26 (1.09, 1.47)	**.002***
**Hypertension**	1.67 (1.45, 1.91)	**<.001***	1.77 (1.39, 2.26)	**<.001***	1.51 (1.30, 1.75)	**<.001***
**Heart failure**	1.36 (1.06, 1.73)	**.02***	1.31 (0.85, 2.03)	.22	1.31 (1.01, 1.69)	.05

© 2024 Chu YY et al. *JAMA Network Open.*

**Atrial fibrillation and flutter**	1.36 (1.08, 1.7)	**.01***	1.35 (0.91, 2.02)	.14	1.22 (0.96, 1.56)	.11
**Ischemic heart diseases**	1.18 (0.98, 1.41)	.08	1.17 (0.84, 1.61)	.35	1.16 (0.96, 1.40)	.13
**Chronic kidney disease**	1.20 (0.97, 1.50)	.10	1.33 (0.90, 1.94)	.15	1.26 (1.00, 1.60)	.05
**Tobacco use**	1.60 (1.29, 2.00)	**<.001***	1.06 (0.69, 1.64)	.79	1.72 (1.35, 2.18)	**<.001***
**OSA**	1.17 (0.89, 1.54)	.27	0.96 (0.58, 1.61)	.88	1.21 (0.9, 1.63)	.21
**Alcohol related disorders**	1.10 (0.71, 1.69)	.67	1.04 (0.47, 2.32)	.92	1.14 (0.71, 1.82)	.58
**Nonruptured cerebral aneurysm**	1.51 (0.87, 2.62)	.14	1.35 (0.50, 3.67)	.56	1.40 (0.76, 2.58)	.28
**Occlusion and stenosis of cerebral arteries**	1.30 (0.51, 3.31)	.58	1.12 (0.21, 6.09)	.90	1.31 (0.48, 3.54)	.60
**Occlusion and stenosis of precerebral arteries**	1.77 (1.34, 2.33)	**<.001***	1.16 (0.67, 2.01)	.59	1.99 (1.49, 2.64)	**<.001***
**DAVF**	1.08 (0.41, 2.89)	.87	1.02 (0.18, 5.78)	.98	1.07 (0.37, 3.09)	.90
**AVM of cerebral vessels**	1.16 (0.004, 330)	.96	1.13 (0, 13,986)	.98	1.07 (0.001, 937)	.98
**Other malformations of cerebral vessels**	1.15 (0.004, 303)	.96	1.13 (0, 12,089)	.98	1.07 (0.001, 849)	.98
**Vitamin B12 deficiency**	1.05 (0.49, 2.28)	.90	1.03 (0.25, 4.25)	.97	1.06 (0.48, 2.38)	.88
**Disorders of sulfur-bearing amino-acid metabolism**	1.03 (0.21, 4.99)	.97	1.02 (0.06, 17.32)	.99	1.01 (0.19, 5.50)	.99
**Morbid obesity**	0.95 (0.67, 1.34)	.78	0.94 (0.51, 1.75)	.85	1.01 (0.69, 1.46)	.98

Abbreviations: *OR*, Odds ratio; *CI*, Confidence interval; *OSA,* Obstructive sleep apnea; *AVM,* Arteriovenous malformation; *DAVF,* Dural arteriovenous fistula

[a] The 10-year OR analysis was restricted to patients who had a decade of follow-up post-diagnosis, with deceased patients still contributing to the analysis.

[b] The adjusted ORs for the occurrence of all strokes, ischemic strokes, and hemorrhagic strokes within one and ten years after NAION were calculated, each accompanied by 95% CIs.

***Statistically significant at *p* < 0.025 after the *Bonferroni* correction.**

eTable 3. Baseline characteristic of NAION cohort and cataract control among patients without comorbidities before and after PSM

	**Before PSM, No (%)**			**After PSM, No (%)**		
	**NAION cohort (n=42,546)**	**Cataract control cohort (n=416,628)**	**SMD**	**NAION cohort (n=29,927)**	**Cataract control cohort (n=29,927)**	**SMD**
**Age at index (mean ± SD)**	37.7 +/- 25.8	65.6 +/- 12.2	**1.39***	50.0 +/- 20.1	50.0 +/- 19.9	<0.001
**Gender (%)**						
**Female**	24,057 (56.5)	248,259 (59.6)	0.06	17,673 (59.1)	16,569 (55.4)	0.08
**Male**	18,317 (43.1)	162,201 (38.9)	0.03	12,093 (40.4)	13,218 (44.2)	0.08

© 2024 Chu YY et al. *JAMA Network Open.*

**Unknown**	172 (0.4)	6,168 (1.5)	**0.11***	161 (0.5)	140 (0.5)	0.01
**Race (%)**						
**American Indian or Alaska Native**	114 (0.3)	951 (0.2)	0.01	72 (0.2)	76 (0.3)	0.003
**Asian**	1,983 (4.9)	12,126 (2.9)	0.09	1,512 (5.1)	1,400 (4.7)	0.02
**Black or African American**	3,352 (7.9)	24,365 (5.8)	0.08	2,175 (7.3)	2,093 (7.0)	0.01
**Native Hawaiian or Other Pacific Islander**	67 (0.2)	1,217 (0.3)	0.03	47 (0.2)	47 (0.2)	0.001
**Other[a]**	2,145 (5.0)	17,081 (4.1)	0.05	1,341 (4.5)	1,401 (4.7)	0.01
**White**	21,955 (51.6)	201,999 (48.5)	0.06	14,692 (49.1)	14,789 (49.4)	0.01
**Unknown**	12,930 (30.4)	158,889 (38.1)	**0.16***	10,088 (33.7)	10,122 (33.8)	0.002
**Ethnicity (%)**						
**Hispanic or Latino**	3,506 (8.2)	15,946 (3.8)	**0.19***	1,844 (6.2)	1,860 (6.2)	0.002
**Not Hispanic or Latino**	28,419 (66.8)	207,940 (49.9)	**0.35***	19,302 (64.5)	19,454 (65.0)	0.01
**Unknown**	10,621 (25.0)	192,742 (46.2)	**0.46***	8,781 (29.3)	8,613 (28.8)	0.01
**Co-medication (%)**						
**Antiplatelet agents**	1,008 (2.4)	11,665 (2.8)	0.03	940 (3.1)	925 (3.1)	0.003
**Anticoagulant agents**	919 (2.2)	7,320 (1.8)	0.03	665 (2.2)	580 (1.9)	0.02
**Antilipemic agents**	1,107 (2.6)	18,753 (4.5)	**0.10***	1,082 (3.6)	1,040 (3.5)	0.01
**Oral Hypoglycemic agents**	351 (0.8)	2,896 (0.7)	0.02	320 (1.1)	321 (1.1)	<0.001
**Insulin**	260 (0.6)	1,338 (0.3)	0.04	214 (0.7)	204 (0.7)	0.004
**Alpha blockers**	290 (0.7)	5,088 (1.2)	0.06	283 (0.9)	301 (1.0)	0.01
**Beta blockers**	1,768 (4.2)	22,669 (5.4)	0.06	1,628 (5.4)	1,922 (6.4)	0.04
**Calcium channel blockers**	616 (1.4)	7,801 (1.9)	0.03	582 (1.9)	546 (1.8)	0.01
**Diuretics**	1,459 (3.4)	15,225 (3.7)	0.01	1,152 (3.8)	1,064 (3.6)	0.02
**ACE inhibitors**	466 (1.1)	7,966 (1.9)	0.07	449 (1.5)	437 (1.5)	0.003
**Angiotensin II inhibitors**	405 (1.0)	6,680 (1.6)	0.06	397 (1.3)	398 (1.3)	<0.001

Abbreviations: *NAION,* Non-Arteritic Anterior Ischemic Optic Neuropathy; *PSM,* propensity score matching; *SD,* standard deviation; *SMD,* standardized mean difference; *ACE*, Angiotensin-converting enzyme.
*** Statistically significant at SMD > 0.1.**
[a] Includes mixed race

© 2024 Chu YY et al. *JAMA Network Open.*
