# Supplementary material for: Stroke Risk Following Nonarteritic Anterior Ischemic Optic Neuropathy
Source: JAMA Netw Open. 2024 Nov 12;7(11):e2444534. doi: 10.1001/jamanetworkopen.2024.44534 (PMC11558474; doi:10.1001/jamanetworkopen.2024.44534)
Supplement: Supplement 2. — Data Sharing Statement [file jamanetwopen-e2444534-s002.pdf]

Data Sharing Statement

Chu. Stroke Risk Following Nonarteritic Anterior Ischemic Optic Neuropathy. *JAMA Netw Open*. Published November 12, 2024. doi:10.1001/jamanetworkopen.2024.44534

Data
Data available: No
